# Root-commando operation for a ruptured left ventricular outflow tract

**DOI:** 10.1093/icvts/ivac036

**Published:** 2022-03-03

**Authors:** Hidenobu Takaki, Kenichi Hashizume, Koki Ikebata, Masatoshi Ohno

**Affiliations:** Department of Cardiovascular Surgery, Saiseikai Utsunomiya Hospital, 911-1 Takebayashicho, Utsunomiya, Tochigi, 321-0974, Japan; Department of Cardiovascular Surgery, Saiseikai Utsunomiya Hospital, 911-1 Takebayashicho, Utsunomiya, Tochigi, 321-0974, Japan; Department of Cardiovascular Surgery, Keio University, 35 Shinanomachi, Shinjuku-ku, Tokyo, 160-8582, Japan; Department of Cardiovascular Surgery, Keio University, 35 Shinanomachi, Shinjuku-ku, Tokyo, 160-8582, Japan

**Keywords:** Root-commando, Aortic root replacement, Left ventricular outflow tract rupture, Prosthetic valve endocarditis, Reoperation

## Abstract

Prosthetic valve endocarditis, especially when complicated by an aortic root abscess and a left ventricular outflow tract rupture, is a life-threatening condition. We present a case of infective prosthetic aortic valve endocarditis with a ruptured left ventricular outflow tract successfully treated with a root-commando operation using a secure anastomosis for reconstruction of the damaged aortic annulus.

## INTRODUCTION

David *et al.* reported aortic and mitral valve operations in combination with intervalvular fibrosa (IVF) reconstruction in cases of infective endocarditis, which came to be named “commando operations” [1].

We present a case of infective aortic prosthetic valve endocarditis (PVE) with a ruptured left ventricular outflow tract (LVOT) treated with a root-commando operation using a secure anastomosis for the reconstruction of the damaged aortic annulus.

## CASE REPORT

**Figure 1:**
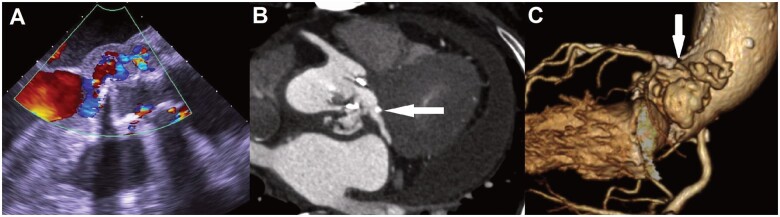
(**A**) Preoperative transoesophageal echocardiography showed the abscess with blood flow in the intervalvular fibrosa. (**B, C**) Preoperative computed tomography demonstrated the ruptured left ventricular outflow tract with contrast extravasation behind the aortic root and ascending aorta (arrow).

A 78-year-old man complaining of pleuritic chest pain and fever was admitted to our hospital. He had undergone aortic valve replacement surgery for aortic valve stenosis 9 years prior to admission. Blood cultures revealed the growth of *Streptococcus anginosus*. Transoesophageal echocardiography showed no perivalvular regurgitation but indicated echo-free space with blood flow in the IVF, which revealed an abscess and bioprosthetic valve endocarditis (Fig. 1A). Computed tomography (CT) showed a ruptured LVOT with contrast extravasation behind the aortic root and ascending aorta (Fig. 1B and C). Magnetic resonance imaging of the patient’s head showed multiple cerebral infarctions with slight bleeding, which were believed to be caused by septic emboli. We diagnosed the patient with infective PVE with a ruptured LVOT and multiple cerebral infarctions and decided to perform an emergency operation.

**Figure 2:**
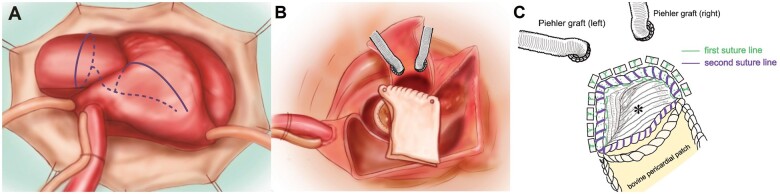
(**A**) Operative incision lines. (**B**) A bovine pericardial quadrangular patch was sewn onto the anterior portion of the bioprosthetic valve cuff. (**C**) Reconstruction of the left ventricular outflow tract with a secure anastomosis using the graft insertion technique. * Reversed artificial graft inserted into the left ventricular outflow tract.

Before resternotomy, the right femoral artery and vein were exposed. Cardiopulmonary bypass was initiated once the chest was entered. As seen on the preoperative CT scan, there was a pseudoaneurysm behind the ascending aorta. Myocardial protection was achieved with antegrade and retrograde blood cardioplegia. The previous bioprosthetic aortic valve was removed, and a defect was found in the LVOT tissue, which was believed to be the entrance of the pseudoaneurysm. The pseudoaneurysm was full of infected blood clots, which were postoperatively confirmed as positive for *Streptococcus anginosus*. The dome of the left atrium also had infected tissue with an IVF abscess. Because the infection seemed to have spread to the subaortic curtain and the muscular interventricular septum and it was necessary to perform radical debridement, we decided to perform a root-commando operation with LVOT reconstruction. After radical debridement of the infected aortic annulus, aortic wall and pseudoaneurysm, the left atrial roof was opened using an extended trans-septal approach (Fig. 2A). Depending on the shape of the root after reconstruction, the distance of the coronary arteries may be insufficient; therefore, we anastomosed an 8-mm graft to the ostium of both coronary arteries to obtain extra mobility. The anterior leaflet of the mitral valve was excised with an IVF abscess, and the posterior leaflet was preserved. Then, 2–0 monofilament sutures were placed on the posterior mitral annulus. Before implanting a 25-mm bioprosthetic valve, a bovine pericardial quadrangular patch, folded in the middle, was sewn onto the remainder of the valve cuff. The posterior bovine pericardial patch was used to replace the left atrial roof (Fig. 2B).

Next, the 30-mm graft was sewn deep in the LVOT using the graft insertion technique [2]. Due to infected and extremely fragile muscular tissue, the reversed graft was double sutured to the LVOT. Subsequently, the graft was sutured to the trimmed anterior bovine pericardium (Fig. 2C), and the reversed graft was pulled out from the outflow tract. The composite graft, consisting of a 30-mm graft and a 25-mm bioprosthetic valve, was sewn onto the graft anastomosed to the LVOT. By setting the suture line higher, additional hemostatic sutures could be easily performed and interference between valves could be avoided. The grafts sewn to the left and right coronary ostia were anastomosed to the composite graft (Video). Weaning from cardiopulmonary bypass was relatively uncomplicated.

**Video:**
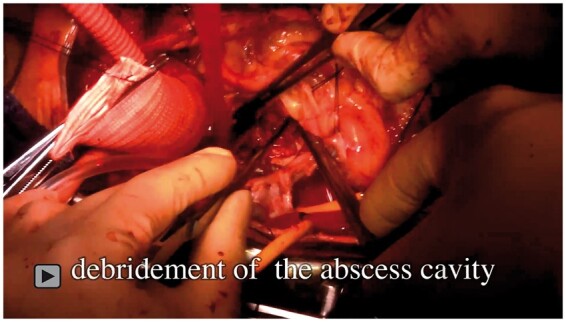
Operative technique of the root-commando operation.

The patient’s postoperative course was stable and uneventful. His heartbeat converted to sinus rhythm on the second postoperative day, and he was extubated on the seventh postoperative day. He was discharged without complications after 6 weeks of postoperative intravenous antibiotic therapy (ampicillin). Postoperative echocardiography and CT showed a well-reconstructed aortic root, IVF and mitral valve. Fifteen months have passed since the operation, and he is currently leading an unrestricted life without complications or evidence of recurring infection.

## DISCUSSION

Aortic root abscess is a life-threatening complication. Leontyev *et al.* reported that 30-day postoperative mortality was 35.5% for patients with PVE with aortic root abscess; the postoperative endocarditis recurrence rate was 8.7% with a mean duration of 1.8 ± 2.4 years [3].

According to the report, the key to complete recovery is to reduce early postoperative mortality and prevent recurrent infection. A root-commando operation with LVOT reconstruction is a technically demanding procedure, but it has the advantages of radical debridement and reconstruction of widely damaged structures. Nakamura *et al.* reported a novel graft insertion technique that provides a secure anastomosis for a damaged LVOT [2].

In conclusion, even in such a devastating condition, with aortic annulus destruction and ruptured LVOT, the root-commando operation with the graft insertion technique could be a lifesaving method. However, further studies are needed to validate the effectiveness of this technique.
